# The Role of NF-κB in Uterine Spiral Arteries Remodeling, Insight into the Cornerstone of Preeclampsia

**DOI:** 10.3390/ijms22020704

**Published:** 2021-01-12

**Authors:** Maciej W. Socha, Bartosz Malinowski, Oskar Puk, Mateusz Wartęga, Martyna Stankiewicz, Anita Kazdepka-Ziemińska, Michał Wiciński

**Affiliations:** 1 Department of Perinatology, Gynecology and Gynecologic Oncology, Faculty of Health Sciences, Collegium Medicum in Bydgoszcz, Nicolaus Copernicus University, Łukasiewicza 1, 85-821 Bydgoszcz, Poland; oskar.trebacz@gmail.com (O.P.); wartega@gmail.com (M.W.); martyna.stankiewicz@cm.umk.pl (M.S.); anita.kazdepka@cm.umk.pl (A.K.-Z.); 2 Department of Obstetrics and Gynecology, St. Adalbert’s Hospital in Gdańsk, Copernicus Healthcare Entity, Jana Pawła II 50, 80-462 Gdańsk, Poland; 3 Department of Pharmacology and Therapeutics, Faculty of Medicine, Collegium Medicum in Bydgoszcz, Nicolaus Copernicus University, M. Curie 9, 85-090 Bydgoszcz, Poland; bartosz.malin@gmail.com (B.M.); wicinski4@wp.pl (M.W.)

**Keywords:** nuclear factor kappa B, preeclampsia, pregnancy, inflammation, pathway, trophoblast, uterine natural killer cells

## Abstract

Preeclampsia is one of the three leading causes of maternal morbidity and mortality worldwide. It afflicts 2–8% of pregnancies and is the most common cause of gestational hypertension. This article is focused on nuclear factor kappa B (NF-κB), its role in normal and pathological spiral arteries remodelling and development of preeclampsia, with evaluation if it is a promising therapeutic target. NF-κB is a key mediator of placentation. Since insemination, it stimulates production of proinflammatory cytokines by the uterine epithelium, which leads to activation of macrophages, uterine natural killer cells (uNKs), and other leukocytes. The trophoblast/uNK/macrophage crosstalk is crucial for implantation and spiral arteries remodeling, and NF-κB regulates that process through modification of cytokine expression, as well as cell phenotype and function. In the course of preeclampsia, the remodeling processes is disturbed by excessive inflammation and increased NF-κB activation. The pathological remodeling leads to uteroplacental dysfunction, release of proinflammatory cytokines into the maternal circulation, endothelial stress, and development of preeclampsia. The analysis of genetic and environmental inductors of NF-κB helps to distinguish preeclampsia risk groups. Furthermore, a selective inhibition of NF-κB or NF-κB activating pathways alleviates symptoms of preeclampsia in rat models; therefore, this could be an efficient therapeutic option.

## 1. Introduction

In 2019, maternal disorders afflicted approximately 112 million females aged 10–54 worldwide and were responsible for 196,000 deaths, and pregnancy induced hypertension (PIH) was the cause of 14% of them [1,2]. PIH, which afflicted approximately 18 million women in 2019, is determined as systolic blood pressure >140 mmHg and/or diastolic blood pressure >90 mmHg and is related with increased risk of cerebrovascular events, disseminated intravascular coagulation (DIC), organ failure, and placental abruption, which are grave complications of pathological pregnancies [3,4]. One of leading causes of PIH and related maternal morbidity and mortality is preeclampsia (PE) [1,2,3]. PE is defined as de-novo hypertension, present after 20 weeks of gestation, combined with proteinuria (>300 mg/day), other maternal organ dysfunction, haematological complications, uteroplacental dysfunction, or foetal growth restriction [3]. Importantly, preeclampsia is considered to be associated with greater risk of DIC, HELLP syndrome, liver hemorrhage, placental abruption, pulmonary oedema, and stroke occurrence among pregnant women [3,4,5]. Since 1990, the incidence of maternal disorders decreased by 8.7%, while the incidence of PIH decreased by 30% [1,2]. This is a notable improvement in healthcare; however, PE is still a great problem, and one of the most common causes of death in obstetrics due to the lack of knowledge about its pathomechanism and no causative treatment. Thereby, many scientists devoted their clinical and scientific careers to addressing these problems. On of results of this strive is this article, in which we delineated the potential pathomechanism of preeclampsia, designated plausible PE triggers, its environmental and genetic factors, as well as potential therapeutic targets. The key element for these issues appears to be the nuclear factor kappa B (NF-κB), which plays a substantial role in normal and pathological placentation, especially spiral arteries remodeling. The aim of this article is to shed light on the role of NF-κB in PE development, which may facilitate future studies and allow for the development of an effective PE treatment.

## 2. Two-Stage Mechanism of PE Development

For decades, scientists have acknowledged the two-stage placental model of preeclampsia [6]. Stage 1, also termed pre-clinical, was characterized by poor placentation, abnormal development of uteroplacental circulation, and no clinical symptoms. These processes are associated with intensive inflammatory response in the uterus. In the Stage 2, also termed the clinical, uterine-originated proinflammatory cytokines diffuse into the maternal circulation in such an amount that they induce systemic inflammation, as well as maternal endothelium dysfunction and as a result hypertension, proteinuria, and finally, eclampsia [6]. Lines of evidence regarding PE pathomechanism advanced through the years, and authors reported that there are many environmental factors, not associated with the placenta, which can initiate systemic inflammation and PE development [6,7]. These factors such as monosodium urate, cholesterol, high concentrations of sodium or glucose, through activation of NF-κB and NOD-like receptor family, pyrin domain-containing protein 3 (NLRP3) inflammasome, can lead to development of PE; sometimes without any observable pathologies in the placenta [7]. However, recently staff revised the two-stage placental model of PE and later, updated models of PE development and stated that the “maternal preeclampsia” does not exist and even if environmental risk factors occur, they lead to PE through alteration of placenta structure and function or overlaps with pre-existed placental pathology, and preeclampsia is always associated with placental disfunction [6]. Whether there are cases of such “maternal preeclampsia” or not, impaired placentation and pathological spiral arteries remodeling plays a major role in development of PE and understanding these processes is an important task for scientists and clinicians, as it would allow for better diagnosis and treatment of many patients. Within the last decade, scientists made a substantial progress regarding this topic, and a key element in comprehension of PE development appears to be the NF-κB.

## 3. Nuclear Factor Kappa B

NF-κB was first identified as a transcription factor regulating expression of immunoglobulin light chain kappa in lymphocytes B; however, soon it was discovered that it controls transcription of many other genes, and after over 30 years of research, over 150 of them can be named [8]. NF-κB is a family of five eukaryotic transcription factors, p50, p52, p65 (RelA), c-Rel, and RelB, encoded by NFKB1, NFKB2, RELA, REL, and RELB genes, respectively [8,9,10]. These proteins do not act solitarily, but form 15 different homodimers and heterodimers, which are translocated into the nucleus and bind to a specific set of related 9–11 bp DNA sites, collectively called kB sites [8]. All proteins have the N-terminal Rel homology domain, which is responsible for both dimerization and binding to κB sites [9]. However, only RelA, RelB, and c-Rel have the transcription activation domain (TAD), which is essential for positive regulation of gene expression [9]. Therefore, p50 and p52 homodimers, as they lack TADs, may downregulate expression of genes with κB sites until they are replaced by NF-κB dimers with TADs. This is only a small part of complexity of gene expression regulation by NF-κB, which allows it to interfere with more than 150 of them. Interestingly, the N-terminal part of Rel homology domain slightly differs between members of NF-κB family, which results in preferential formation of heterodimers rather than homodimers [11]. Furthermore, Rel homology domains make base-specific and non-specific contacts with DNA, and aforementioned variations result in different affinity to particular genes, depending on number of their DNA base pairs (9–11) in κB sites [11].

In resting cells, NF-κB is stored in the cytoplasm in its inactive form as dimers associated with inhibitors of nuclear factor kappa B (IκB) or precursor proteins p100 and p105, which prevents NF-κB from entering into the nucleus [8,9,10]. Proteins p100 and p105 are precursor proteins of p52 and p50, respectively, and if cleavaged to their active form they facilitate transportation into the nucleus and DNA binding. There are three typical types of IκB proteins, IκBα, IκBβ, and IκBε, and three types of inducibly expressed, atypical IκB, Bcl-3, and IκBζ [8,9,10]. Every NF-κB has the nuclear localization sequence (NLS), which facilitates its transportation into the nucleus; however, it is covered by IκBs [9,10]. Therefore, activation of NF-κB requires its release from IκB, and predominantly this process is mediated by a complex of IκB kinases (IKK), IKKα, IKKβ, and IKKγ, which leads to phosphorylation, ubiquitination, and degradation of IκB by proteasome [10].

There are three known NF-κB activation pathways: canonical, non-canonical, and atypical [10,11,12,13].

The canonical pathway leads to activation of NF-κB through degradation of IκBs by IKK complex [10,11,12]. The activators of this pathway are interleukin 1 (IL-1) and a vast number of different damage-associated molecular patterns (DAMPs) and pathogen-associated molecular patterns (PAMPs), such as high-mobility group box 1 (HMGB1), fibrinogen, extracellular DNA, ATP and histones, glycans, flagellin, lipoteichoic acid, etc. IL-1 is a ligand for IL-1 receptors (IL-1Rs) and activated IL-1R recruits the interleukin-1-receptor-associated kinase (IRAK-1) through the adapter molecule—myeloid differentiation primary response 88 (MYD88) [14,15,16]. Recruited IRAK-1 is highly phosphorylated and dissociates form the receptor into the cytoplasm and associates with IKKα–IKKβ–IKKγ complexes, where it activates IKKγ, also termed the NF-κB essential modulator (NEMO). Activated NEMO oligomerize and initiate autophosphorylation of IKKs, hereby activating the complex [9,14,15]. Active IKK complex phosphorylates and leads to degradation of IκBs, therefore, leads to release and activation of NF-κB. Interestingly, active IRAK-1 is also translocated into the nucleus, where it associates with promoters of NF-κB regulated genes increasing their expression, inter alia, the expression of IκBα [14]. Thereby, IRAK-1 mediates not only the activation of NF-κB, but also regulates its inhibition. Other receptors involved in the NF-κB activation are toll-like receptors (TLRs), whose ligands are DAMPs and PAMPs, and the whole process is identical, as in the case of IL-1Rs [14].

The non-canonical pathway can be activated by the tumor necrosis factor (TNF), B-cell activating factor (BAFF), CD40 ligands, or virus RNA [9,12,14]. Activated BAFF receptor (BAFFR), CD40, lymphotoxin β receptor (LTβR), or receptor activator for NF-κB (RANKL) associates with TNF receptor associated factors (TRAFs) complexes, which in turn release the NF-κB-inducing kinase (NIK) [17]. NIK activates IKKα, which forms homodimers and promotes IKKα association with p100 in p100/RelB heterodimers. Then NIK phosphorylates p100, which leads to its ubiquitination and processing in proteasome into p52 [17]. Active p52/RelB heterodimer is translocated into the nucleus where it regulates gene expression.

The atypical pathway is not one mechanism of NF-κB activation, but rather a term used for any of them that is different from canonical and non-canonical pathways and which is often not fully comprehended [12,13]. However, most often it involves casein kinase 2 (CK2), which is activated by reactive oxygen species, hypoxia, UV, or DNA damage phosphorylase IκBs, leading to its degradation in proteasome and release of NF-κB [12,13]. However, the exact mechanism of this process is unknown.

The variety of NF-κB activating factors, activation pathways, and regulated genes contribute to the complexity of NF-κB signaling and its involvement in different processes. One of them is a remodeling of uterine spiral arteries during pregnancy, and the crucial role of NF-κB in that process is described in the following section.

## 4. NF-κB as a Mediator of Spiral Arteries Remodeling

### 4.1. General Information

Considering that NF-κB is an essential mediator for many processes, it is no surprise that it appears to be a key regulator during pregnancy, taking part in its development, maintenance, and termination. Within the first days after the conception, NF-κB regulates time of the implantation through induction of leukemia inhibitory factor (LIF) production and inflammation, which results in removal of mucins covering adhesion molecules present on the surface of uterine epithelium [18,19]. Furthermore, inflammation is associated with increased expression of L-selectin on the surface of uterine epithelial cells, facilitating blastocyst adhesion and implantation [18]. Inflammation was reported to be characteristic for the implantation period, and the first trimester of pregnancy in general is associated with increased production of many signaling molecules, such as TNFα, chemokine (C-X-C motif)-ligand 1 (CXCL1), IL-1, IL-2, IL-8, and IL-15, whose expression is controlled by NF-κB [8,20,21,22,23]. Mor et al. indicated that embryo implantation presents “an open wound” phenotype, which further increases NF-κB activation [22]. Inflammatory state in uterus stimulates local white blood cells such as dendritic cells and uterine natural killer cells (uNK) and also leads to infiltration of the implantation site by new monocytes, lymphocytes, and neutrophils [24,25]. Interestingly, inflammation, associated activation of leukocytes, and initial changes in spiral arteries have been reported to occur before the implantation; therefore, embryo antigens are not initiators of inflammation in the uterus, which is crucial for embryo adhesion and implantation [26,27]. Researchers hypothesized that this is a result of hormonal changes during the menstrual cycle; however, an important trigger for uterine inflammation appear to be insemination [26,27].

### 4.2. Insemination as an Inflammation Trigger

Semen is not simply a carrier of spermatozoa, but it has various functions like increase of pH, facilitating the movement of spermatozoa, etc. [26,27,28]. For decades, it has been the subject of research, and knowledge of its role in fertilization and embryo development has grown steadily. First trials with in vitro fertilization in rodent models showed that embryo transfers to females not exposed to male fluids were associated with greater rates of implantation failure, miscarriage, foetal growth restriction, and abnormalities [29,30]. Similar findings have been made regarding human embryos [31,32]. Therefore, investigation of seminal fluid components followed.

Many studies reported that semen induces inflammatory response in the uterus through specific mediators and presentation of paternal antigens [26,27,28,33]. The most important seminal cytokine is transforming growth factor β (TGFβ), which stimulates uterine epithelial cells to production of proinflammatory cytokines, such as granulocyte-macrophage colony stimulating factor (GM-CSF), colony stimulating factor 1/macrophage colony stimulating factor (CFS1)/(M-CSF), IL-1α, IL-6, IL-8, LIF, RANTES, and macrophage inflammatory protein 1α (MIP-1α) [26,27,28]. This process is most likely mediated through activation of the TGFβ receptor I and II (TGFβRI/TGFβRII) complex, which in turn leads to the activation of NF-κB; however, this process is not fully comprehended [34]. Torrealba et al. examined the expression of TGFβ, TGFβRI, TGFβRII, NF-κB, PI3K, AKT, and mTOR in specimens collected from 106 patients with prostatic cancer [35]. Analysis revealed increased expression of TGFβ, PI3K/AKT/mTOR pathway compounds, IKK, and NF-κB [35]. Relating these findings to previous studies, researchers assumed that TGFβ through TGFβRs activates the PI3K/AKT/mTOR pathway, which in turn activates IKK complexes, thus NF-κB [35,36]. This process is possibly responsible for induction of proinflammatory cytokine production by uterine epithelium, as GM-CSF, M-CSF, IL-1α, IL-6, IL-8, LIF, RANTES, and MIP-1α gene expression is regulated by NF-κB [8,19]. Robertson et al. indicated that these mediators induce inflammation in uterus and activate uterine dendritic cells (DCs) and macrophages, and the latter leads to further stimulation of DC and inflammation spreading through TNFα release [27]. Moreover, aforementioned cytokines lead to substantial recruitment and activation of circulating monocytes and granulocytes [27]. Activated DCs have an important role, as they act as antigen presenting cells (APCs) presenting paternal MHC antigens to lymphocytes T, which results in their hypo-responsiveness for paternal antigens and fetal cell immunotolerance [27,33]. As was mentioned in the previous section, inflammation leads to removal of mucins covering epithelium and presentation of adhesion molecules e.g., L-selectin [18]. Moreover, infiltrating macrophages and neutrophils produce various metalloproteinases (MMPs), inter alia MMP-2 and MMP-9, which disintegrate the extracellular matrix (ECM) in order to facilitate implantation and trophoblast invasion [37,38]. Interestingly, it has been reported that MMP-9 production in breast cancer cells in regulated by PI3K/AKT/NF-κB pathway; therefore, it can be assumed that MMP-9 release by macrophages and neutrophils is also controlled by NF-κB [39]. These findings indicate that NF-κB is a key mediator of the implantation process. Alongside macrophages, uterine natural killer cells (uNKs) are the most prominent type of leukocytes in the placental bed [40]. They are activated by HLA class I antigens, HLA-C, HLA-E, and HLA-G presented by trophoblast and regulate spiral arteries remodeling and placentation [40,41,42,43,44]. The trophoblast invasion and uNK activation starts a sophisticated crosstalk between macrophages, uNKs, and trophoblasts, on which the future of pregnancy depends.

### 4.3. Trophoblast/uNK/Macrophage Crosstalk

Uterine NKs are different from circulating NKs and are called CD56bright, as they show increased expression of CD56 [45]. This type of NKs was reported to be less cytotoxic and to produce more cytokines than other NK types; thus, it was assumed that CD56bright have a regulatory function in inflammation [40,43,45]. In a mouse model, Chkraborty et al. have proven that the presence of uNKs is crucial for spiral arteries remodeling [46]. Uterine NK cell depletion was achieved by treatment with anti-asialo GM1 antibodies on gestation day 4.5 or 4.5 and 9.5. Placentation sites were collected and examined on day 9.5 or 13.5 [46]. The disruption of the vascular smooth muscle layer of spiral arteries was observed only in samples with uNKs and was abolished in samples lacking uNKs [27]. Furthermore, trophoblast invasion into the uterine wall was much deeper and chaotic in uNK deficient uteri [46]. Interestingly, uNKs modified trophoblast phenotype led researchers to conclusions that uNKs control disorganization of spiral arteries’ smooth muscle layers, modulate trophoblast phenotype, and restrain its invasion [46]. Zhang and Tian proposed a mechanism of uNK function in which killer-cell immunoglobulin-like receptors expressed by uNK recognize MHCs presented by trophoblasts [43]. This leads to the activation of uNKs and production of various factors, such as IL-8 and CXCL-10, which in turn affect trophoblasts through CXCR1 and CXCR3 receptors, and this is most likely the mechanism of trophoblast phenotype and function modification by uNKs [29]. In response to that, stimulation trophoblasts produce TGF-β, chorionic gonadotropin (HCG), and VEGF [43]. The other factors produced by activated uNKs are M-CSF, GM-CSF, TNF-α, IFN-γ, TGF-β1, VEGF, PlGF, LIF, angiopoietin 1 (Ang-1), Ang-2, MMP-2, and MMP-9 [40,43,47,48]. These data indicate that uNKs and trophoblasts enhance the embryo related inflammatory response in uterus, leading to further activation of uNKs and trophoblasts. Liu et al. reported that TGF-β1 has a direct impact on vascular smooth muscle cells (VSMCs) through maternally expressed gene 3 (MEG-3) and induces VSMCs apoptosis and migration and suppresses their proliferation [44]. These effects of TGF-β1 were abolished in MEG-3 silenced VMSC cultures [44].

**Figure 1:**
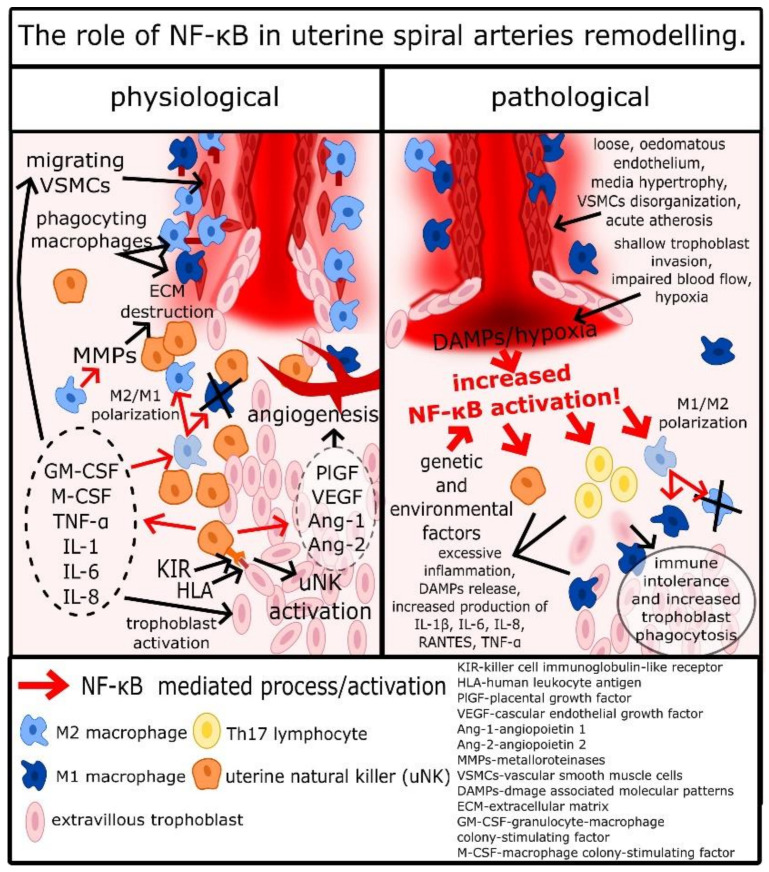
The role of NF-κB in uterine spiral arteries remodeling.

Other cells important for spiral arteries remodeling and placentation are macrophages. Some authors indicated that in the remodeling process, macrophages M2 are the majority of macrophages infiltrating the spiral arteries site; however, it appears that these macrophages are hard to define, as there can be many intermediate types between proinflammatory type M1 and anti-inflammatory M2 [49]. It is most likely a result of a great variety of aforementioned signaling factors associated with implantation related inflammation. For example, TGF-β, IL-6, and VEGF are simulating M2 polarization, while TNF-α and IFN-γ are stimulating M1 polarization, and combination of these factors may result in formation of atypical macrophages [50,51]. Nevertheless, it appears that there are more M2 macrophages in uterus during spiral arteries remodeling [49,52]. Macrophages activated by cytokines and other factors produced by endometrium epithelial cells, uNKs, and trophoblasts infiltrate decidua around spiral arteries and secrete a wide range of cytokines, IL-1β, IL-4, IL-6, IL-8, IL-10, IL-13, and TNF-α, which lead to further extension of inflammatory response and stimulation of uNKs [49,52]. However, the most important function of macrophages is the secretion of MMP-1, MMP-2, MMP-7, MMP-9, and MMP-10, disorganization of the extracellular matrix, and phagocytosis of apoptotic VSMCs and trophoblasts [40,49,52,53]. Although it was reported that macrophages have no impact on VSMCs migration and apoptosis, the disorganization of the extracellular matrix facilitates that process, and phagocytosis of post-apoptotic cell rests decreases DAMP release, which alongside with secretion of IL-10 restrains excessive inflammatory response, which could dysregulate the remodeling process [40,49,52,53]. As it was mentioned before, uNKs secrete TNF-α and IFN-γ, which are essential to stimulate uterine macrophages to produce CXCL10 [54]. Another CXCL10 production stimulator is hypoxia, which frequently occurs in the uterus during the placentation [55,56]. In such environment macrophages, infiltrating spiral arteries produce CXCL10, which is a potent chemoattractive molecule for trophoblasts [57]. Therefore, during the placentation, macrophages not only facilitate but also stimulate spiral arteries invasion by trophoblasts. Finally, monocytes stimulated by GM-CFS differentiate into dendritic cells (DCs), which through secretion of soluble FMS-like protein 1 (sFlt-1) and TGF-β1 play a pivotal role in placentation related angiogenesis [50,58]. As a result of the described processes, VSMCs detach from the spiral arteries’ wall, undergo apoptosis, and become phagocytosed by macrophages, while extravillous trophoblasts take their place in the spiral artery wall [25]. This process, strictly controlled by trophoblast/uNK/macrophage crosstalk, results in the formation of wide, cone-like endings of spiral arteries, in which maternal blood flows around foetal capillaries and exchanges oxygen, carbon dioxide, nutrients, etc. [25]. This complex and fragile process is a basis of pregnancy; its disturbance can have dread consequences and is regulated by the trophoblast/uNK/macrophage subtle crosstalk as it is summarized in Figure 1.

### 4.4. The Role of NF-κB in Trophoblast/uNK/Macrophage Crosstalk

Like it was described above, NF-κB has an important role from the very beginning of the pregnancy, as it regulates the expression of cytokines by epithelial cells, through the TGF-β1/TGF-βRs/PI3K/AKT/mTOR/IKK/NF-κB pathway [34,35,36]. Therefore, placentation starts with NF-κB regulated production of GM-CSF, M-CSF, IL-1α, IL-6, IL-8, LIF, RANTES, and MIP-1α by uterine epithelial cells. KIR receptors present on the surface of NKs have TLR-like properties, and Rajagopalan et al. show that association of KIR-2DL4 with HLA-G leads to activation of NF-κB [59,60]. Therefore, activation of uNKs by trophoblasts leads to the release of proinflammatory cytokines by uNKs in the NF-κB-dependent mechanism [61]. Decidual macrophages mostly have the M2 phenotype, and this type of cell has anti-inflammatory properties necessary for limitation of inflammation in uterus and regulation of spiral arteries remodeling [18]. Yunna et al. reported that macrophage polarization into the M2 phenotype is regulated by NF-κB, where increased NF-κB activation promotes M1 differentiation and decreased activation promotes M2 differentiation [62]. Chang et al. shed light on that process, describing that activation of TLR-2 leads to activation of p105/p65 heterodimers and p52/p65 increases the expression of p65 [63]. However, p65 in this mechanism, after transportation into the cytosol, is ubiquitinated and aggregated with other ubiquitinated p65 [63]. Such aggregates are internalized by autophagosomes and destructed [63]. Therefore, TLR2 activation in macrophages leads not to the activation of NF-κB, but to the decrease of p65 concentrations. According to Yu et al. among TLR2 ligands are hialuronian, endoplasmin, and high-mobility group protein 1 (HMGB1); therefore, the destruction of the extracellular matrix, cell apoptosis, and pyroptosis associated with spiral arteries remodeling and inflammation in the uterus can promote M2 polarization of decidual macrophages through decrease of NF-κB, which is possibly the inflammation self-restrain process, very important in regulation of implantation [64]. Regardless of the type of macrophages, NF-κB was reported to upregulate secretion of MMP-2 and MMP-9, which are crucial for disorganization of the extracellular matrix and trophoblasts invasion [21]. As was mentioned before, hypoxia occurs frequently during placentation and development of the placenta, and it stimulates angiogenesis. Hypoxia is an important NF-κB activator, and it was reported that in this mechanism, trophoblasts are stimulated to produce VEGFs, such as PlGF, which are critical for proper development of arteries in the placenta [21,65]. Interestingly, not only does the PlGF gene have NF-κB sites, but the endoglin gene and endoglin are also a part of the TGFβRs complex [62]. Finally, expression of many cytokines secreted by trophoblasts, uNKs, and macrophages, such as GM-CSF, M-CSF, TNF-α, IL-1α, IL-1β, IL-6, and IL-8, are regulated by NF-κB [8,20,21]. Therefore, NF-κB is a key mediator of placentation and spiral arteries remodeling, and disturbance of its regulatory function leads to a pathological development of pregnancy.

### 4.5. Abnormal Placentation in PE and the Role of NF-κB

During the physiological placentation, trophoblasts differentiate into villous cytotrophoblasts and syncytiotrophoblast, which form placenta villi and extravillous trophoblasts, which invade uterine decidua and take part in spiral arteries remodelling [24,25]. These extravillous trophoblasts infiltrate spiral arteries and replace VSMCs, forming wide, cone-like endings of these vessels, with a gentle widening. With progression of pregnancy, these trophoblasts are covered with endothelial cells, and such prepared vessels allow maternal blood to flow around placental capillaries and exchange oxygen, metabolites, etc., between mother and foetus [25]. Extravillous trophoblasts migrate against the blood flow, and some of them form a conglomerate in the proximal part of artery, resulting in transient hypoxia [24]. Hypoxia, alongside various cytokines, is an important mediator of spiral arteries remodeling, as was described in the section on trophoblast/uNK/macrophage crosstalk. It stimulates the invasion of extravillous trophoblasts into the arteries, but also helps to limit it and curb the extension of the remodeling to the distal end of the artery, as well as to slow down this process and make it steady [24,25].

Histological analysis of placenta samples acquired from women with PE revealed a significant impairment of spiral arteries remodeling, termed decidual vasculopathy [66,67]. Spiral arteries of women with PE were often characterized by loose, edematous endothelium, hypertrophy of the media, disorganization of the smooth muscle layer, and acute atherosis [66,68]. Stanek investigated placenta samples acquired from 230 women with early-onset PE, 261 women with late-onset PE, and 5059 healthy women [67]. Preeclampsia, regardless of the onset, was associated with increased occurrence of decidual arteriopathy, chronic hypoxic placental injury, villous infarction, membrane laminar necrosis, membrane microscopic chorionic pseudocysts, clusters of maternal floor multinucleated trophoblasts, excessive number of extravillous trophoblasts, and intervillous thrombi, in comparison to healthy individuals [67]. Chronic hypoxic placental injury was more common in early-onset PE than late-onset PE and correlated with more severe clinical outcomes [67] In PE, extravillous trophoblasts either infiltrate the uterine wall chaotically and deeper than normal or, as occurs more frequently, the invasion of trophoblasts is too shallow, limited only to the very end of arteries [21,24,25]. In this situation, there are very wide endings of arteries with significant narrowing right before them [24]. In such conditions, blood flows with greater pressure and mechanically widens aforementioned lacunas and also provides less oxygen than physiological, gently widening spiral arteries [5,21,24,25]. Moreover, stagnation of blood in lacunas leads to increased exposition of surrounding cell, inter alia trophoblasts, free radicals, harmful metabolites, cytokines, etc., and can lead to formation of clots [5,24,25]. This results in further, pathological intensification of inflammation, whilst hypoxia/reperfusion and excessive inflammation appear to be the main causes of the pathological remodeling [21,24,25]. To support that hypothesis, it should be mentioned that similar effects of hypoxia on blood vessels were observed in other tissues. For example, Kitchen et al. indicated that hypoxia leads to blood vessel remodeling in the blood–brain barrier, causing its function alteration and central nervous system oedema [69]. Therefore, it is very likely that the remodeling role of hypoxia is consistent throughout the human body, including the placenta.

In the case of PE, the aforementioned pathological inflammatory response is clearly mediated by NF-κB, as its abnormal elevation is characteristic for this disease [21]. High amounts of IL-1β, IL-6, IL-8, RANTES, and TNF-α, whose expression is regulated by NF-κB, leads to trophoblasts apoptosis, release of DAMPs, and further activation of NF-κB [8,21,24]. The aforementioned hypoxia/reperfusion insult leads to increased cell apoptosis and necrosis, release of DAMPs and PAMPs, and activation of NF-κB, as was described in Section 3, and if these processes exceed abilities of decidual macrophages to phagocyte cell rests/DAMPs and “tide-up” the surrounding of spiral arteries, the remodeling process is disturbed [21,24]. Therefore, inflammation and NF-κB, which regulates production of proinflammatory cytokines and angiogenic factors, activation of macrophages, uNKs, and trophoblasts, are crucial for physiological placentation and spiral arteries remodeling; however, they must be carefully balanced through the pregnancy, as excessive activation of NF-κB leads to pathological placentation. There is still no clearly defined cause of the excessive activation of NF-κB and pathological inflammatory response in the uterus, although there are some interesting studies on this topic.

## 5. Risk Factors and Genetic Ground of Preeclampsia

In view of the described studies, excessive inflammation in the uterus leads to the pathological spiral arteries remodeling and abnormal function of the placenta and contributes to the development of preeclampsia. As it was proven in the previous section, the NF-κB is a key mediator of inflammatory response in the course of both physiological and pathological placentation. However, to fully comprehend the process of PE development, some important questions must be addressed, specifically, what triggers an excessive inflammatory response, is NF-κB signaling disturbance the source of this problem or only its mediator, and could NF-κB targeted therapy alleviate pathological remodeling and prevent PE occurrence?

According to the modified two-stage model of preeclampsia presented by Staff, PE risk factors are both genetic and environmental [6]. The genetic component is particularly pronounced in cases of early-onset PE, while the environmental component is more often associated with late-onset PE [6]. It was reported that the presence of preeclampsia in first degree relatives increases a woman’s risk of PE 2–4 fold [70]. Furthermore, not only the maternal but also the paternal genotype may contribute to PE development, as there is a higher risk of PE if the male fathered a preeclamptic pregnancy with a different partner or was born of a pregnancy complicated with PE [70]. This led authors to the conclusion that the pathological inflammatory response could be a result of immunological intolerance of foetal tissues, most likely trophoblasts. This process could be related with excessive activation of NF-κB and inflammation, infiltration of the uterus by NF-κB-induced Th17 cells, which also express KIRs [20,71]. Moreover, western blot analysis performed by Ridley et al. showed that KIR-3DL2 expression is regulated by NF-κB [71]. As was described before, uNKs through KIRs recognize trophoblasts; however, uNKs are generally secretory, not cytotoxic, but Shields et al. showed that ischemia-induced Th17 may mediate polarization of NKs toward a cytotoxic phenotype [72]. Therefore, maybe physiological inflammation and hypoxia in uterus during placentation facilitate recognition of specific HLA by Th17, which mediate further progression of inflammation and induction of cytotoxic NKs, although this is only a hypothesis. However, the analysis of genetic polymorphisms of maternal KIR and foetal HLA-C in 484 normal and 254 pre-eclamptic pregnancies performed by Nakimuli et al. revealed that some KIR variants are significantly more common among women with PE, some do not reveal association with PE, and some even are less common among women with PE than among healthy individuals [73]. The combination of maternal KIR AA genotype and fetal HLA-C2 alleles is associated with higher risk of PE occurrence, with odds ratio (OR) = 1.49, *p* = 0.0318, *n* = 724 for Uganda cohort and OR = 1.46, *p* = 0.0267, and *n* = 1321 in the United Kingdom cohort [73]. This would indicate that maternal–foetal KIR-HLA incompatibility is a major aspect in the PE pathomechanism; however, studies on larger groups of patients are needed.

Another interesting study was conducted by Sakowicz et al., who analyzed variants of the NEMO gene among 72 preeclamptic women, 79 controls, and their children [74]. This gene is located on the chromosome X, and two different variants termed A and T were distinguished. Statistical analysis revealed that occurrence of the TT genotype in both mother and children (TT in a female child, T in a male child) were related, with a higher likelihood of preeclampsia development (OR = 2.59, *p* < 0.05) and increased NEMO activity [74]. This could be a result of increased half-time of NEMO mRNA; therefore, its up-regulated expression and higher concentrations of NEMO can possibly lead to more frequent and extended multimerization of IKK complexes, which facilitates their activation. However, the exact mechanism is unknown.

According to the modified two-stage model of PE, other, nongenetic factors, such as cholesterol, monosodium urate, etc., can activate NF-κB, stimulate inflammation, and lead to development of PE [6].

In summary of this section, different inductors of inflammation appear to be triggers for excessive inflammatory response, abnormal placentation, and PE development, and it is hard to analyze and predict all risk factors. Considering that mechanism, inhibition of the inflammation, e.g., through inhibition of NF-κB, seems to be a reasonable therapeutic strategy; however, NF-κB balances anti-inflammatory and proinflammatory responses in the uterus, and any involvement in the beginning of the pregnancy can disturb that complex and fragile process.

## 6. Perspectives

Considering a crucial role of NF-κB in the pathological remodeling of spiral arteries and development of preeclampsia, assessment of its elevation, and analysis of mother’s exposure to NF-κB activating factors or processes could be very helpful in distinguishing the PE risk groups and better management of high-risk pregnancies. NF-κB targeted therapy could be an efficient treatment of PE; however, this transcription factor is very important in physiological placentation; therefore, there is a risk of disorganization of this process with NF-κB inhibition. Thereby, it must be very carefully considered and balanced to keep NF-κB activation in physiological range. Further studies with assessment of the risk–-benefit ratio of this type of treatment will be of great value for this topic.

An interesting approach would be a selective inhibition of TLR4, as Nizyaeva et al. demonstrated that TLR4 expression is significantly higher among women with PE in comparison to healthy controls [75]. Researchers analyzed syncytiotrophoblasts and endothelium of intermediate villi acquired from 37 women aged 23–40 years who were divided into four groups: early-onset PE (EPE; 12 patients), late-onset PE (LPE; 10 patients), late normal (LN; 10 women with physiological gestation curse), and early normal (EN; five women with premature operative labor at 26–31 week gestation). The TLR4 expression was significantly higher in the EPE group in comparison to the EN group (21.0 ± 2.3 vs. 13.0 ± 1.8, *p* = 0.00001) [75]. Moreover, expression of toll interacting protein (Tollip), which is an inhibitory adaptor protein for TLRs, was higher in PE groups in comparison to controls [75]. Although it is a promising study, it has some limitations, like low number of enrolled patients and that the difference between TLR4 expression achieved statistical significance only between EPE and EN groups, and the differences in Tollip expression were statistically significant only between EPE and LPE [75]. However, this may be a result of too small a number of samples, and analysis within a larger group should follow. Nevertheless, in rat models, inhibition of TLR4 and as a result, inhibition of NF-κB resulted in alleviation of PE symptoms in rat models of LPS-induced PE [76,77]. Therefore, supplementation of Tollip or treatment with different TLR4 inhibitors could be an efficient therapeutic option for preeclampsia.

Excessive inflammatory response in uterus, pathological placentation, and remodeling of spiral arteries can be dangerous for pregnancy due to a risk of placental insufficiency, foetal growth restriction, or even placental abruption [6,7]. However, maternal symptoms of PE are the effect of systemic inflammation, which often originates in uterus but not necessarily [6,7]. Therefore, inhibition of this pathological inflammatory response with drugs with non- or low penetration into the foetal part of placenta and blood probably would reduce the risk of disturbance of beneficial NF-κB mediated processes and would ameliorate maternal symptoms. Eddy et al. created an elastin-like polypeptide (ELP), associated it with NF-κB inhibitor p50i, and administered it to pregnant rats [78]. Fusion of p50i with ELP prevented its placental transfer in pregnant rats, resulting in increased deposition in the maternal kidney, liver, and placenta relative to the free peptide [78]. Furthermore, in a rat model of placental ischemia, ELP-p50i partially ameliorated placental ischemia-induced hypertension and reduced placental TNF-α levels with no signs of toxicity [78].

An interesting approach in order to further investigate spiral arteries remodeling and PE pathomechanism could be a creation of organoids. Lancaster and Huch assessed recent studies in this matter, concluding that organoids can be created from human stem cells, that these constructs imitates architecture of certain tissues, e.g., endometrium, and can be used as a reliable source of information about particular processes in certain tissues in in-vitro studies [79]. Furthermore, Turco et al. created an endometrial organoid and exposed it to pregnancy signals such as prolactin and human chorionic gonadotropin, which resulted in organoid morphology change into a decidua-like characteristic for placentation [80]. Such in vitro models of early pregnancy can facilitate studies on placentation and associated pathologies, like PE.

Another development, which can ease investigation of PE pathomechanism, was made by Salman et al., who created an microvessel-on-chip open microfluid model [81]. This model allows to create in vitro microvasculature from endothelial cells and control fluid flow through these vessels, and most importantly, observe these vessels with high resolution imaging modalities, such as electron microscopy, fluorescence imaging, etc. [81]. If it will be able to apply this method to the spiral arteries model, it could allow for real time observation of spiral arteries remodeling, which is a key process in placentation and, if pathological, in PE development.

This study shows how important NF-κB is in the pathomechanism of PE, and if further positive findings follow, this could be a beginning of a new chapter in treatment of preeclampsia.

## 7. Conclusions

NF-κB is a key mediator of embryo invasion, spiral arteries remodeling, and development of the placenta. NF-κB mediates production of proinflammatory cytokines by the uterine epithelium, which activates decidual macrophages. In these macrophages, NF-κB stimulates the production of MMPs, which are necessary for disorganization of the extracellular matrix. Moreover, NF-κB regulates M1/M2 polarization of macrophages, or through GM-CFS, their differentiation into dendritic cells. Inter alia through these processes, NF-κB mediates a self-restriction of the inflammatory process and immunotolerance of foetal cells, like trophoblasts. NF-κB plays a crucial role in the trophoblast/uNK/macrophage crosstalk, which is necessary for proper invasion of trophoblasts and spiral arteries remodeling. Disturbance of this sophisticated crosstalk and increased activation of NF-κB leads to an excessive inflammatory response, pathological placentation, and in consequence may lead to uteroplacental dysfunction, release of proinflammatory cytokines into the maternal circulation, endothelial stress, and development of preeclampsia. The excessive activation of NF-κB can be environmental or genetic. In some cases, it involves immune intolerance of foetal antigens, in which NF-κB may play an important role as an inductor of Th17 lymphocytes and KIR-3DL2 expression. Genetic analysis revealed that a certain combination of maternal and foetal NEMO gene polymorphism is associated with increased risk of PE. Moreover, some combinations of KIR and HLA alleles are related with increased, while some with decreased, risk of PE development. Analysis of both environmental and genetic factors in view of NF-κB may allow for better distinction of PE risk groups. Increased activation of NF-κB appears to be crucial for pathological processes associated preeclampsia and selective inhibition of NF-κB alleviated PE symptoms and reduced placental impairment in rat models. Both inhibition and activation of NF-κB must be well balanced for physiological placentation and pregnancy development; therefore, therapies proposed in rat models must proceed with a careful safety analysis. However, this type of treatment may be a beginning of a new chapter in treatment of preeclampsia.
